# Body iron stores had no impact on coronary heart disease outcomes: a middle-aged male cohort from the general population with 21-year follow-up

**DOI:** 10.1136/openhrt-2021-001928

**Published:** 2022-04-10

**Authors:** Salim Bary Barywani, Erik Östgärd Thunström, Zacharias Mandalenakis, Per-Olof Hansson

**Affiliations:** 1 Department of Molecular and Clinical Medicine, Sahlgrenska Academy, University of Gothenburg, Medicine, Gothemburg, Sweden; 2 Department of Medicine, Geriatrics and Emergency Medicine/Östra, Sahlgrenska University Hospital, Region VästraGötaland, Medicine, Gothenburg, Sweden

**Keywords:** Myocardial Infarction, Percutaneous Coronary Intervention, Epidemiology, Coronary Artery Disease

## Abstract

**Background:**

Body iron stores (BISs) have been proposed to be related to the development of cardiovascular diseases. However, results from epidemiological studies are conflicting. Knowledge on the long-term impact of BIS on cardiovascular outcomes in the general population is lacking.

**Purpose:**

The aim of this study was to explore the relationship between BIS and coronary heart disease (CHD) including death due to CHD.

**Methods:**

This investigation is part of ‘The Study of Men Born in 1943’, a longitudinal prospective study of men living in the city of Gothenburg, Sweden. This random population sample was examined in 1993 (all at 50 years of age at baseline). A medical examination was performed, and questionnaires were used to evaluate lifestyle factors. Biomarkers for iron stores (serum ferritin and serum transferrin receptor) was analysed from frozen blood samples in 2014. All hospital admissions were registered through national registers during the entire follow-up from 1993 to 2014. HRs were estimated by Cox proportional-hazard regression analyses.

**Results:**

During the 21 years follow-up period, 120 participants (15.2%) developed CHD and 16 patients (2%) died due to CHD. The all-cause mortality was 15.2% (n=120) including 40 cardiovascular deaths (5.1%). In a multivariable Cox regression analysis, the daily smoking, hypertension and the increased resting heart rate was independent predictors of CHD, while no significant association was found between BIS and risk of CHD.

**Conclusions:**

In a cohort of middle-aged men from the general population with well validated and prospectively collected data, we did not find any association between serum ferritin or serum transferrin receptor as markers of BIS and CHD events after 21 years of follow-up.

**Trail registration number:**

NCT03138122.

**Key questions**

**What is already known about this subject?**

- Epidemiological data on iron status and cardiovascular disease risk are conflicting, and the long-term impact of the body iron stores (BIS) on the coronary heart disease (CHD) outcomes in the general population is still lacking.

**What does this study add?**

- The 21-year incidence of CHD in middle-aged men from general population was as high as about 15%.
- BIS markers: serum ferritin and serum transferrin receptor had no relationship with the risk for coronary heart disease events.

**How might this impact on clinical practice?**

- This study support the theory that BIS has no relationship with cardiovascular risk.
- Further studies are needed regarding the relationship between the intake of red meat and increased cardiovascular risk.

## Introduction

The impact of increased body iron stores (BIS) as a risk factor for cardiovascular events has been debated during the last decades. It has been reported that in addition to the traditional risk factors (such as hypertension, hyperlipidaemia, diabetes, obesity, low physical activity level and smoking), the BIS might play an important role in the pathogenesis of atherosclerosis and cardiovascular outcomes.^1–10^ Furthermore, the different mechanisms during which the iron influences the pathogenesis of atherosclerosis and hence increased risk for major adverse cardiovascular events have also been described.^1 6–11^ Previous studies have also showed the relationship between BIS and destabilisation of atherosclerotic plaque.^10 11^ Depending on the available evidence, it has been proposed that limiting meat consumption should be added to the list of traditional healthy lifestyle recommendations.^4 8 12 13^ For instance, Snowdon *et al*^8^ as early as the 1980s demonstrated a 60% increase in the risk of fatal coronary artery events among men who consumed meat six times a week compared with men who consumed meat less than once a week, and Salonenn *et al*^9^ in a cohort of Finnish men, demonstrated that the risk of coronary heart disease (CHD) was significantly associated with iron intake. In contrast, other studies have discarded the association between iron stores and atherosclerosis.^5 14 15^ For instance, Zacharski *et al*^5^ in a randomised clinical trial demonstrated that the reduction of BIS by regular phlebotomy at 6-month intervals over a 6-year period had no effect on all-cause mortality, cardiovascular death, non-fatal myocardial infarction or stroke in patients with peripheral vascular disease. Epidemiological data on iron status and cardiovascular disease risk are accordingly conflicting, and the long-term impact of the BIS on the CHD outcomes in the general population is still lacking. This study aimed to explore the relationship between BIS and the cardiovascular incidence in terms of CHD in a general male population during 21 years of follow-up.

## Methods

### Study design and setting

The ‘Study of Men Born in 1943’ is a longitudinal, prospective cohort study initiated in 1993 investigating cardiovascular risk factors and diseases. As there are several papers deriving from the same study population there are limited different ways to describe the same methodology. We prefer to describe the method used for collecting the baseline data in a standardised way.^16^ A randomly selected sample of one-half of all men born in 1943 and living in the city of Gothenburg, Sweden, were invited to participate. Of 1463 men invited, 798 (55%) accepted participation and underwent a health examination at entry. Participants who had a CHD event before the study start (n=13) were excluded from the analyses. Altogether, 785 participants were included in the analyses.

### Data collection

At the baseline examination, medical history of each participant was obtained, and a physical examination was performed. In the 2014 visit, all study participants also underwent an echocardiography examination. Fasting venous blood samples were drawn before clinical examinations 1993. A panel of biomarkers was analysed (Elecsys, Roche) from blood samples drawn at the visit in 1993 and kept frozen (−70°C) until analysis in 2014. The analysis included biomarkers for BIS serum ferritin, and serum transferrin receptor (sTfR), N-terminal prohormone of brain natriuretic peptide, high-sensitive C reactive protein (CRP), Interleukin 6 (IL-6), creatinine, cystatin C, total serum cholesterol, triglycerides and high-density lipoprotein. Blood pressure was recorded in the right arm in the sitting position by a mercury sphygmomanometer. Participants considered having hypertension or diabetes mellitus if they had an established diagnosis. Body mass index (BMI) was calculated as weight (kg) divided by height squared (m^2^). Before the examination, all participants had completed questionnaires addressing their smoking habits, physical activity, previous diseases and mental stress. Participants were classified as never smokers, previous smokers or current smokers. Physical activity during leisure time was introduced at each examination and graded as follows: (1) mainly sedentary, (2) moderate exercise during leisure time, (3) regular exercise and training, (4) hard exercise or competitive sports. For analysis, grades 2, 3 and 4 were combined and grade 1 was defined as a sedentary lifestyle. Mental stress, with six response options, was defined as feeling tense, irritable or filled with anxiety or having sleeping difficulties as a result of conditions at work or at home: (1) never experienced stress, (2) one period of stress ever, (3) some periods in the past 5 years, (4) repeated periods during the past 5 years, (5) permanent stress during the past year and (6) permanent stress during the past 5 years, with 5–6 defined as mental stress.

### Follow-up procedures and endpoints

All participants were followed from baseline in 1993 to 31 August 2014. Outcome and clinical data were collected from the Swedish Hospital Discharge Registry and the Swedish Death Registry for all participants from 1993 to 2014. The major endpoints were CHD events including new onset myocardial infarction, treatment with percutaneous coronary intervention (PCI), treatment with coronary artery bypass grafting CABG and death due to coronary artery disease. All endpoints were reviewed by one of five physicians, all specialists in cardiology or internal medicine. The cohort was divided to two groups with comparison between participants with S-ferritin below and over 350 ng/mL. This cut-off had been chosen because the available data have considered serum ferritin levels 200–350 as limits for body iron overload^17^ (table 1).

### Statistics

Categorical variables were described as percentages and compared using χ^2^ test or Fisher’s exact as appropriated. Continuous variables were tested for the normality of the distribution using visual inspection of their histograms and normal QQ plots. The continuous variables were described as means±SD and compared using independent t-test. Correlation analysis for the relationship of the S-ferritin and sTfR with each other and their relationship with the inflammatory markers was performed. To adjust for the underlying clinical conditions and to analyse for probable association between BIS and CHD, the cohort was analysed using Cox proportional-hazard regression models analysing time to the first endpoint event. The HRs were adjusted for the atherosclerotic cardiovascular disease risk factors including also the lifestyle related factors, socioeconomic status and cardiac biomarkers. Systematically and according to the principles of Cox-regression model building the results were adjusted for all the baseline variables demonstrated in table 1. The high event rate (120 events) gave the opportunity to adjust for the all-baseline variables (table 1). Thereby, all baseline variables in table 1 were included in the univariable models, (online supplemental table 1). Variables associated with CHD with a (p <0.25) in the univariable models were included in the multivariable models. Variables associated with CHD with a (p >0.25) in the univariable models were according to the principles of model building also tested in the multivariable models. The multivariable Cox models were assessed for proportional hazard assumption for covariates graphically with adjusted log minus log curves. HRs with CIs and p values are presented. Although, the multivariable Coxproportional-hazard regression models were adjusted for the inflammatory markers (CRP and IL-6) a further Cox proportional-hazard regression analysis was performed, excluding participants with CRP >10 (n=18). All statistical analyses were performed using SPSS V.22 statistical software. A p<0.05 was regarded as significant.

10.1136/openhrt-2021-001928.supp1 Supplementary data

**Table 1:** The baseline characteristics at 50 years of age, comparing participants with S-ferritin below and over 350 ng/mL, n=785

Baseline variables	Total cohort Mean±SD. N (%)	S-ferritin <350 ng/mL Mean±SD. N (%)	S-ferritin >350 ng/mL Mean±SD. N (%)	P value
Estimated glomerular filtration rate, mL*/*min*/*1.73 m^2^. n=713	102±17	101±17	106±18	0.020
Body mass index, kg/m2, n=713	26±3.4	26±3.3	28±3.8	<0.001
Systolic blood pressure, mm Hg, n=710	129±17	127±17	136±18	<0.001
Diastolic blood pressure, mm Hg, n=709	88±11	87±11	91±12	<0.001
Resting heart rate, bpm, n=713	67±12	66±14	73±11	0.001
Total cholesterol, mmol/L, n=712	5.9±1.0	5.9±1.0	6.0±0.9	0.142
Fasting plasma glucose, mmol/L, n=710	4.5±0.8	4.5±0.7	5.0±1.3	0.086
C reactive protein, mg/mL, n=713	2.2±3.6	2.1±3.4	3.1±5.0	0.161
Interleukin-6, pg/mL, n=710	2.8±4.0	2.6±2.2	2.5±1.4	0.140
NTproBNP, ng/L, n=713	33±39	33±38	34±49	0.397
High sensitive troponin T, ng/L, n=713	5±3.3	5.5±3.2	5.5±4.4	0.455
Hypertension, n=712	83 (11.6)	62 (10.2)	21 (20.3)	0.003
Regular moderate physical activity for a minimum 3 hours a week, n=713	604 (85)	519 (85)	85 (82.5)	0.630
Daily smoker, n=713	221 (30)	202 (33.1)	19 (18.4)	0.008
Experienced several periods of stress or more the last 5 years, n=710	111 (16)	94 (15.4)	17 (16.5)	0.535
More than 10 years formal education, n=712	336 (47.2)	288 (47.3)	48 (46.6)	0.830

NTproBNP, N-terminal prohormone of brain natriuretic peptide.

## Results

### Baseline characteristics

The baseline characteristics are presented in table 1. The cohort had a mean BMI (26 kg/m^2^), 85% reported regular moderate physical activity for a minimum 3 hours a week, 30% were smokers and 16% had experienced several periods of stress or more the last 5 years. Compared with participants with S-ferritin <350, participants with S-ferritin >350 ng/mL had significantly higher estimated glomerular filtration rate, higher BMI, higher systolic and diastolic blood pressure, higher resting heart rate and had more frequently hypertension diagnosis, but were less frequently daily smoker. There were no statistically significant differences between the two groups regarding 10 year’s formal education, CRP, IL-6, total cholesterol, NT-pro-BNP, highly sensitive troponin T, physical activity and stress levels.

### Outcomes

During 21 years of follow-up, 120 participants (15.2%) developed CHD; 32 participants developed acute ST-elevation myocardial infarction, 55 participants developed non-ST-elevation myocardial infarction, 63 participants underwent PCI, 47 participants underwent CABG and 16 participants (2%) died due to CHD. The all-cause and cardiovascular death were 120 (15.2%) and 40 (5.1%), respectively. Of the non-cardiovascular deaths, 50 (6.3%) were due to cancer, 23 (2.9%) deaths caused by other identified causes and 7 (0.09%) deaths were due to unknown causes.

### Analyses for association between body iron sources (S-ferritin and sTfR) and CHD events

**Figure 1:**
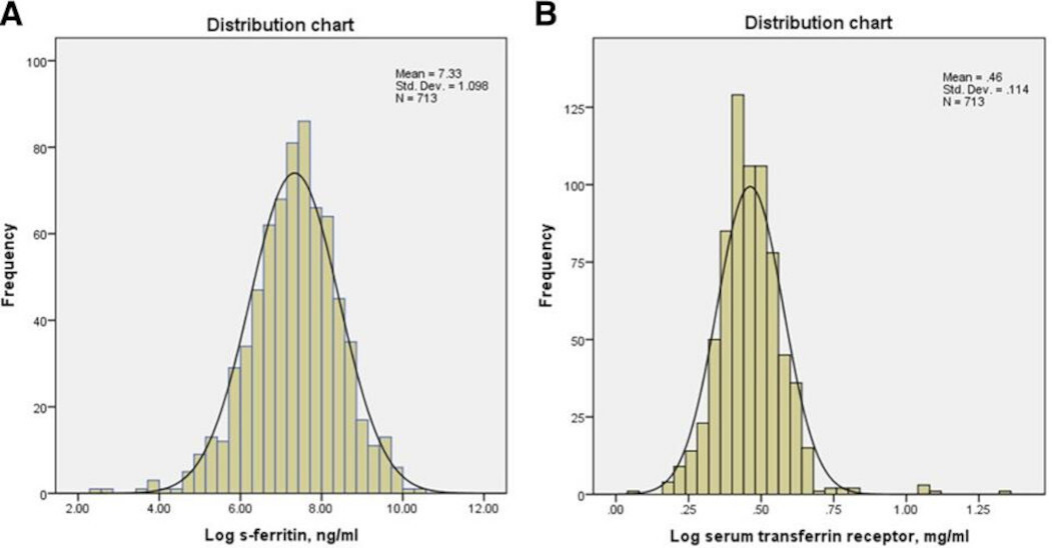
(A) Distribution of log S-ferritin. (B) Distribution of log serum transferrin receptor.

S-ferritin and serum transferrin receptor (sTfR) we reanalysed as continuous variables (figure 1A, B and tables 2 and 3). The high event rate (120 events) gave the opportunity to adjust for the all-baseline variables in table 1. Neither univariable, nor multivariable Cox proportional-hazard regression models did demonstrate any significant association between the composite combined endpoint of CHD events and the S-ferritin or sTfR. Univariable Cox-regression analyses are demonstrated in online supplemental table 1, the multivariable Cox-regression analyses for S-ferritin and sTfR in tables 2 and 3, respectively.

**Table 2:** Multivariable Cox proportional-hazard regression model; relationship between S-ferritin and coronary heart disease events during 21 years of follow-up

	P value	HR	95% CI for HR
Log. s-ferritin, n=712	0.434	0.94	0.82 to 1.09
Estimated glomerular filtration rate >75 mL/min/1.73 m^2^, n= 712	0.801	1.1	0.48 to 2.59
Body mass index, kg/m^2^, n= 712	0.225	1.03	0.98 to 1.08
Regular moderate physical activity for a minimum 3 hours a week, n= 712	0.327	0.82	0.56 to 1.21
Daily smoker, n=712	<0.001	1.78	1.3 to 2.45
More than 12 years formal education, n=712	1.193	0.81	0.59 to 1.11
Resting heart rate, bpm, n =712	0.018	1.01	1.001 to 1.03
C reactive protein >3 mg/L, n=712	0.438	0.855	0.57 to 1.27
NT-pro-BNP>99 ng/L, n= 700	0.338	1.34	0.74 to 2.44
Total cholesterol, mmol/L, n= 712	0.362	1.07	0.92 to 1.24
Hypertension, n=712	0.002	1.94	1.28 to 2.94

NT-pro-BNP, N-terminal prohormone of brain natriuretic peptide.

**Table 3:** multivariable Cox proportional-hazard regression model; relationship between serum transferrin receptor and coronary heart disease events during 21 years of follow-up

	P value	HR	95% CI for HR
Log, serum transferring receptor, n=712	0.279	1.06	0.95 to 1.18
Estimated glomerular filtration rate>75 mL/min/1.73 m2, n=712	0.808	1.1	0.48 to 2.59
Body mass index, kg/m^2^, n=712	0.286	1.02	0.98 to 1.07
Regular moderate physical activity for a minimum 3 hours a week, n= 712	0.341	0.83	0.57 to 1.22
Daily smoker, n=712	<0.001	1.84	1.34 to 2.53
More than 12 years formal education, n=712	0.183	0.81	0.59 to 1.1
Resting heart rate, bpm, n =712	0.021	1.015	1.002 to 1.027
C reactive protein >3mg/L, n=712	0.437	0.85	0.57 to 1.27
NT-pro-BNP>99 ng/L, n=712	0.355	1.33	0.73 to 2.41
Total cholesterol, mmol/l, n=712	0.398	1.07	0.92 to 1.24
Hypertension, n=712	0.002	1.91	1.27 to 2.88

NT-pro-BNP, N-terminal prohormone of brain natriuretic peptide.

### Determinants of increased CHD events

The adjusted Cox proportional-hazard regression multivariable models demonstrated, daily smoking, hypertension and increased resting heart rate as independent predictors of CHD events, (p<0.001, HR 1.78, 95% CI 1.30 to 2.45), (p 0.002, HR 1.94, 95% CI 1.28 to 2.94) and (p 0.018, HR 1.01, 95% CI 1.001 to 1.03), respectively (tables 2 and 3).

### The distribution of the S-ferritin and sTfR and their relationship to each other and with inflammatory markers

**Figure 2:**
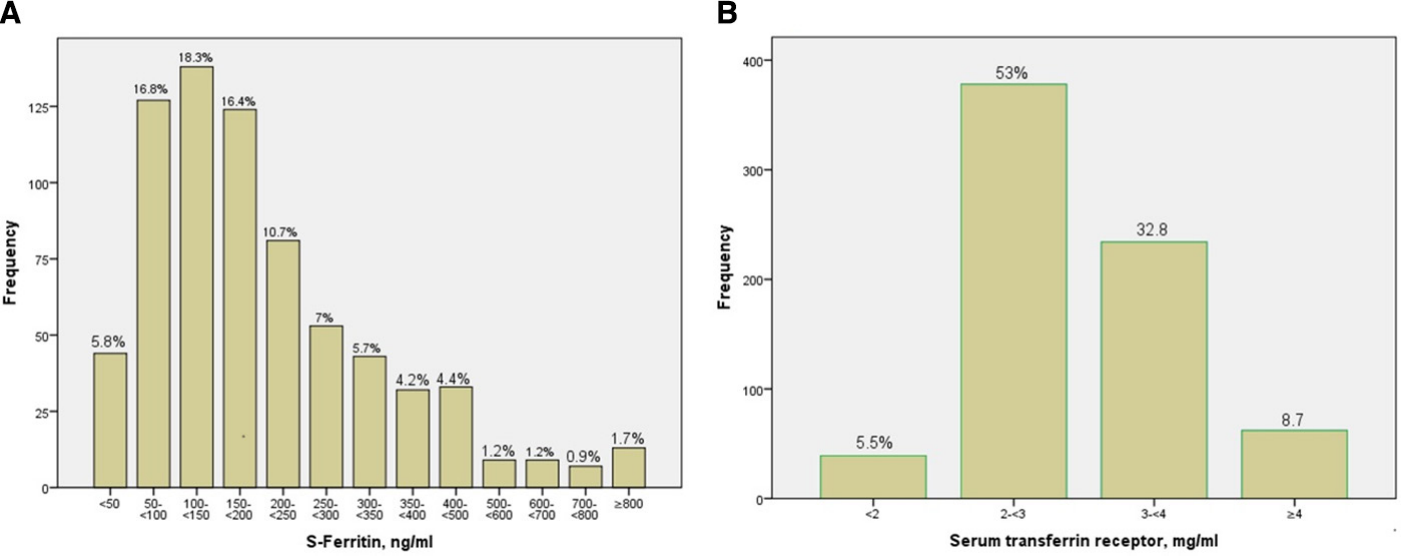
(A) S-ferritin distribution. (B) Serum transferrin receptor distribution.

The distribution of S-ferritin is presented in figure 2A. Seven participants had iron deficiency with S-ferritin below 20 ng/mL and 13 participants had a S-ferritin above 800 ng/mL. Regarding serum transferrin receptor (sTfR), 11 participants had signs of iron deficiency with values above 5 ng/mL, the distribution of sTfR is presented in figure 2B. The data demonstrated a positive correlation between both, CRP and IL6 and S-ferritin, and a negative correlation between S-ferritin and sTfR.

## Discussions

This study with well validated and prospectively collected data and a follow-up of more than two decades did not demonstrate any association between a composite endpoint of CHD and serum ferritin or serum transferrin receptor (sTfR). The statistical analyses were of high power and the risk of a missed association is therefore very low. However, this study demonstrated some traditional risk factors as independent predictors of increased risk for CHD; daily smoking, hypertension and increased resting heart rate. Ferritin is an acute phase reactant protein, and it elevates in the case of infections or inflammations and the serum ferritin levels in these cases might not reflect the real BIS. The data demonstrated a positive correlation between both, CRP and IL-6 and serum ferritin. However, the Cox regression models were adjusted for these two variables. Furthermore, a sensitivity analysis was performed, excluding participants with CRP >10 (n=18) and still no association between serum ferritin and CHD was found. Moreover, the sTfR (a marker for BIS which does not influences by the inflammatory status) was also studied in survival analyses, and. no significant association with the risk for CHD events was detected. It is worthy to mention that the correlation analyses demonstrated a negative correlation between S-ferritin and sTfR indicating that the S-ferritin in the cohort represents the body iron status and probably not influenced by any inflammation. Previous studies demonstrating positive relationship between BIS and increased cardiovascular risk have mostly adjusted for a few potential confounders, and rarely for lifestyle-related factors, socioeconomic status and cardiac biomarkers and none being long-term survival studies.^3 12 13^ For instance, Liu *et al*^3^ included only anthropometric measurements (BMI and waist circumference) and blood glucose measurements. Moreover, most of the studies demonstrating the effect of BIS on cardiovascular risk have studied different mechanisms and pathophysiological explanations rather than longitudinal long-term survival outcomes.^1 6 7 10 11 14 15 18^ A substantial part of these studies has used the dietary intake of iron rather than the BIS as a cardiovascular risk factor.^4 8 12 13^ Associations have, thus, been demonstrated between iron containing foods and cardiovascular outcome rather than the BIS and the outcome. Moreover, contrary to the iron-heart hypothesis, several biomarkers of iron status like serum iron or transferrin saturation have shown robust inverse associations between iron concentrations and cardiovascular risk.^19^ Therefore, the previous data on relationship between the iron body sources and cardiovascular disease might be masked with other aspects of the lifestyle that increase the risk for cardiovascular diseases.^20^ Red meat might exert its negative effect on cardiovascular events through other mechanisms than the irons suspected pathological effects. However, there are available data discarding the association between iron stores and atherosclerosis.^5 19^

## Conclusions

In this cohort of middle-aged men from the general population, we did not find any association between S-ferritin or sTfR as markers of BIS and CHD events after 21 years of follow-up.

### S-ferritin as a marker for BIS

Ferritin is an iron storage protein. It consists of a protein shell which encloses a core of ferric-hydroxy-phosphate which can hold up to 4000 atoms of iron. The major function of ferritin is to provide a store of iron which may be used for haem synthesis when required. With the help of sensitive immunoradiometric assay that ferritin was detected in the serum or plasma of normal individuals. There is a good relationship between the total amount of stored body iron and the serum ferritin concentration in normal individuals.^21^ For the last 30 years, serum ferritin has been an important tool for investigating changes in storage iron concentration. According to the WHO, the serum ferritin level is the most specific biochemical test that correlates with relative total BIS. The guide for programme managers^17^ concluded that thresholds of >200 µg/L for men and >150 µg/L for women were appropriate. In the UK, values of >300 µg/L for men and elderly women have been suggested.^17^

### Strengths and limitations

This study had a well-validated and prospectively collected data giving the opportunity to in addition to the traditional cardiovascular risk factors adjust also for the cardiac biomarkers, inflammatory markers, socioeconomic and lifestyle related risk factors (table 1), with a follow-up of more than two decades. However, the cohort included only men and the results cannot be extrapolated to the middle-aged women. Reference ranges for haemoglobin and serum ferritin in women of reproductive age are widely reported showing values that are lower than equivalent aged males, but gradual increase to a level slightly lower than in men approximately 10 years after menopause.^22 23^ However, Albrektsen *et al* found only minor changes in the incidence rate of MI when moving from menopausal to postmenopausal age.^24^

### Clinical implications

This study supports the theory that BIS has no relationship with cardiovascular risk. However, further studies are needed regarding the relationship between the intake of red meat and increased cardiovascular risk or to find more representative BIS markers.

## Data Availability

Data are available on reasonable request. The data that support the findings of this study are available on request from the corresponding author, SBB.

## References

[1] Kraml P. The role of iron in the pathogenesis of atherosclerosis. Physiol Res 2017;66:S55–67. 10.33549/physiolres.933589 28379030

[2] Tofano RJ, Pescinni-Salzedas LM, Chagas EFB, et al. Association of metabolic syndrome and hyperferritinemia in patients at cardiovascular risk. Diabetes Metab Syndr Obes 2020;13:3239–48. 10.2147/DMSO.S271050 33061489 PMC7522598

[3] Liu J-R, Liu Y, Yin F-Z, et al. Serum ferritin, an early marker of cardiovascular risk: a study in Chinese men of first-degree relatives with family history of type 2 diabetes. BMC Cardiovasc Disord 2019;19:82. 10.1186/s12872-019-1068-5 30943893 PMC6448272

[4] Fang X, Wei J, Min J, et al. Dietary intake of heme iron is associated with increased cardiovascular disease risk: reply to Dr. Bitterman. Nutr Metab Cardiovasc Dis 2020;30:1053–5. 10.1016/j.numecd.2020.03.007 32247532

[5] Zacharski LR, Chow BK, Howes PS, et al. Reduction of iron stores and cardiovascular outcomes in patients with peripheral arterial disease: a randomized controlled trial. JAMA 2007;297:603–10. 10.1001/jama.297.6.603 17299195

[6] Recalcati S, Locati M, Marini A, et al. Differential regulation of iron homeostasis during human macrophage polarized activation. Eur J Immunol 2010;40:824–35. 10.1002/eji.200939889 20039303

[7] Duffy SJ, Biegelsen ES, Holbrook M, et al. Iron chelation improves endothelial function in patients with coronary artery disease. Circulation 2001;103:2799–804. 10.1161/01.CIR.103.23.2799 11401935

[8] Snowdon DA, Phillips RL, Fraser GE. Meat consumption and fatal ischemic heart disease. Prev Med 1984;13:490–500. 10.1016/0091-7435(84)90017-3 6527990

[9] Salonen JT, Nyyssönen K, Korpela H, et al. High stored iron levels are associated with excess risk of myocardial infarction in eastern Finnish men. Circulation 1992;86:803–11. 10.1161/01.CIR.86.3.803 1516192

[10] Butcher MJ, Galkina EV. Phenotypic and functional heterogeneity of macrophages and dendritic cell subsets in the healthy and atherosclerosis-prone aorta. Front Physiol 2012;3:44. 10.3389/fphys.2012.00044 22457649 PMC3307136

[11] Ley K, Miller YI, Hedrick CC. Monocyte and macrophage dynamics during atherogenesis. Arterioscler Thromb Vasc Biol 2011;31:1506–16. 10.1161/ATVBAHA.110.221127 21677293 PMC3133596

[12] Wolk A. Potential health hazards of eating red meat. J Intern Med 2017;281:106–22. 10.1111/joim.12543 27597529

[13] Micha R, Michas G, Mozaffarian D. Unprocessed red and processed meats and risk of coronary artery disease and type 2 diabetes--an updated review of the evidence. Curr Atheroscler Rep 2012;14:515–24. 10.1007/s11883-012-0282-8 23001745 PMC3483430

[14] Auer J, Rammer M, Berent R, et al. Body iron stores and coronary atherosclerosis assessed by coronary angiography. Nutr Metab Cardiovasc Dis 2002;12:285–90. 12616808

[15] Waalen J, Felitti V, Gelbart T, et al. Prevalence of coronary heart disease associated with HFE mutations in adults attending a health appraisal center. Am J Med 2002;113:472–9. 10.1016/S0002-9343(02)01249-4 12427496

[16] Chen X-J, Barywani SB, Hansson P-O, et al. Impact of changes in heart rate with age on all-cause death and cardiovascular events in 50-year-old men from the general population. Open Heart 2019;6:e000856. 10.1136/openhrt-2018-000856 31168369 PMC6519434

[17] WHO, UNICEF, UNU. Iron deficiency anaemia: assessment, prevention, and control. A guide for programme managers, 2001. Available: https://apps.who.int/nutrition/publications/micronutrients/anaemia_iron_deficiency/WHO_NHD_01.3/en/index.html

[18] Grammer TB, Kleber ME, Silbernagel G, et al. Hemoglobin, iron metabolism and angiographic coronary artery disease (the Ludwigshafen risk and cardiovascular health study). Atherosclerosis 2014;236:292–300. 10.1016/j.atherosclerosis.2014.07.002 25112800

[19] Gutierrez-Bedmar M, Olmedo P, Gil F. Low serum iron levels and risk of cardiovascular disease in high-risk elderly population: nested case-control study in the PREvención Con DIetaMEDiterránea (PREDIMED) trial. ClinNutr 2020;12:S0261–5614. 10.1016/j.clnu.2020.05.044 32591250

[20] Zhang Y-B, Chen C, Pan X-F, et al. Associations of healthy lifestyle and socioeconomic status with mortality and incident cardiovascular disease: two prospective cohort studies. BMJ 2021;373:n604. 10.1136/bmj.n604 33853828 PMC8044922

[21] Walters GO, Miller FM, Worwood M. Serum ferritin concentration and iron stores in normal subjects. J Clin Pathol 1973;26:770–2. 10.1136/jcp.26.10.770 4750458 PMC477879

[22] Albrektsen G, Bønaa KH. Body iron stores and the gender gap in risk of incident myocardial infarction-reply. JAMA Intern Med 2017;177:595–6. 10.1001/jamainternmed.2016.9675 28384768

[23] Milman N. Serum ferritin in Danes: studies of iron status from infancy to old age, during blood donation and pregnancy. Int J Hematol 1996;63:103–35. 10.1016/0925-5710(95)00426-2 8867722

[24] Albrektsen G, Heuch I, Løchen M-L, et al. Lifelong gender gap in risk of incident myocardial infarction: the Tromsø study. JAMA Intern Med 2016;176:1673–9. 10.1001/jamainternmed.2016.5451 27617629

