# Supplementary material for: Body iron stores had no impact on coronary heart disease outcomes: a middle-aged male cohort from the general population with 21-year follow-up
Source: Open Heart. 2022 Apr 10;9(1):e001928. doi: 10.1136/openhrt-2021-001928 (PMC9003602; doi:10.1136/openhrt-2021-001928)
Supplement: Supplementary data [file openhrt-2021-001928supp001.pdf]

Supplementary table 1, univariable Cox proportional-hazard regression models, time to first coronary heart disease event, 120 events.

Variables	P-value	HR	95.0% CI for HR	
			Lower	Upper
Log S-Ferritin, ng/ml	0.868	0.988	0.861	1.134
Log. serum transferrin receptor, mg/ml	0.394	1.05	0.94	1.16
Estimated glomerular filtration rate, mL/min/1.73 m^2	0.001	1.009	1.004	1.015
Body mass index, kg/m^2	0.013	1.052	1.011	1.095
Systolic blood pressure, mmHg	0.024	1.009	1.001	1.017
Diastolic blood pressure, mmHg	0.018	1.015	1.002	1.027
Resting heart rate, bpm	0.001	1.025	1.010	1.040
Total cholesterol, mmol/l	0.009	1.202	1.046	1.380
Log. C-reactive protein, mg/ml	0.004	1.180	1.54	1.321
C-reactive protein >1mg/l	0.004	1.597	1.158	2.203
Interleukin-6>5 pg/ml	0.463	1.219	0.718	2.072
Fasting plasma glucose >4.9 mmol/l	0.100	1.368	0.942	1.985
NTproBNP>99 ng/l	0.244	1.417	0.788	2.548
Highly sensitive troponinT> 5 ng/l	0.332	1.164	0.857	1.582
Hypertension	<0.001	2.140	1.686	3.081
Regular moderate physical activity for a minimum 3 hours a week	0.021	0.635	0.432	0.934
Daily smoker	<0.001	2.056	1.538.	2.750
Experienced several periods of stress or more the last 5 years	0.059	1.421	0.987	2.046
More than 10 years formal education	0.012	0.685	0.509	0.921
